# Decreasing myopic lacquer crack and widening parapapillary gamma zone: case report

**DOI:** 10.1186/s12886-021-02216-7

**Published:** 2021-12-24

**Authors:** Rahul A. Jonas, Chuan Chuan Wei, Jost B. Jonas, Ya Xing Wang

**Affiliations:** 1 Department of Ophthalmology, University Hospital of Cologne, Cologne, Germany; 2 Beijing Institute of Ophthalmology, Beijing Tongren Eye Center, Beijing Tongren Hospital, Capital University of Medical Science, Beijing Ophthalmology and Visual Sciences Key Laboratory, 1 Dongjiaomin Lane, Dongcheng District, Beijing, 100730 China; 3 Department of Ophthalmology, Medical Faculty Mannheim, Heidelberg University, Kutzerufer 1, 68167 Mannheim, Germany; 4 Institute of Molecular and Clinical Ophthalmology Basel, Basel, Switzerland; 5 Privatpraxis Prof Jonas und Dr Panda-Jonas, Heidelberg, Germany

**Keywords:** Lacquer crack, Gamma zone, Myopia maculopathy, Pathologic myopia, Case report. High myopia, Parapapillary gamma zone, Parapapillary delta zone

## Abstract

**Background:**

Myopic axial elongation may be due to an equatorial enlargement of Bruch’s membrane (BM), leading to a prolate eye shape and increasing strain with BM and the retinal pigment epithelium (RPE) layer at the posterior pole. The increased BM strain may cause an enlargement of Bruch’s membrane opening (BMO) of the optic nerve head, with the subsequent development and enlargement of parapapillary gamma zone as BM-free parapapillary zone. The increased strain within BM and RPE may also cause lacquer cracks (LCs) as linear breaks in the RPE and / or BM. Studies suggested that a more marked gamma zone enlargement is associated with lower prevalence of LCs or macular BM defects. Here report on the disappearance of a LC during a 10-year follow-up of a highly myopic eye, concurrent with a marked increase in gamma zone.

**Case presentation:**

A 56-year-old woman showed in her right eye (axial length measured 30.69 mm) a LC, vertically oval optic disc, and parapapillary gamma zone in 2001. When re-examined in 2006, gamma zone had enlarged, while the LC was no longer detectable. In 2011, the LC was not visible neither upon ophthalmoscopy and or upon optical coherence tomography (OCT), while gamma zone had further enlarged. The gamma zone enlargement occurred in a direction perpendicular to the direction of the former LC.

**Conclusions:**

The observation suggest that a LC can decrease in width, in temporal association with an enlargement of gamma zone. It fits with the notion that an enlargement of the BMO (i.e., enlarging gamma zone) may lead to a relaxation of the BM strain and subsequently to a decrease in the width of the LC.

## Background

Lacquer cracks (LCs) are linear breaks in the retinal pigment epithelium (RPE) and / or Bruch’s membrane (BM), are usually located at the posterior pole, and are risk factors for progression of myopic maculopathy [1]. In 1988, Klein and Green postulated a mechanical stretching and rupture of the BM-RPE-choriocapillaris complex as cause for LCs [2]. Recent clinical and histological findings supported the notion of BM playing a biomechanical role in the etiology of myopic axial elongation [3]. In the posterior fundus, BM has with the Bruch’s membrane opening (BMO) a physiologic defect which forms the inner layer of the optic nerve head canal. Recent studies suggested that during myopic axial elongation in non-highly myopic eyes, BMO shifts backward, leading to an overhanging of BM into the intrapapillary region of the optic nerve head at the nasal disc border, and an absence of BM in the temporal parapapillary region [4]. That BM-free area has been called gamma zone [3]. With further axial elongation leading to high myopia, BMO enlarges so that the intrapapillarily overhanging part of BM is retracted and a circular gamma zone develops. It has been discussed that the backward shift of the BMO may be caused by a BM enlargement in the equatorial region, and that the BMO enlargement is due to an axial elongation-associated increase in the strain within BM in the posterior fundus region [3]. This increased BM strain has also been regarded as causative for the development of LCs as linear BM defects, eventually progressing to areolar BM defects in eyes with myopic maculopathy. A recent study suggested that a larger BMO in highly myopic eyes was associated with a lower prevalence of secondary macular BM defects [4]. It led to the hypothesis, that a reduction in the BM strain by a sufficiently large increase in the BMO (i.e., a gamma zone enlargement) may prevent the development of additional macular BM defects. Fitting with this hypothesis, we here report on a highly myopic patient who showed a disappearance of a LC during a 10-year follow-up, concurrent with a marked increase in gamma zone.

## Case presentation

**Fig. 1:**
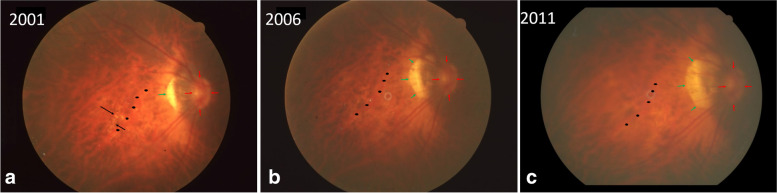
Fundus photograph taken in 2001 (**a**), 2006 (**b**) and in 2011 (**c**) of a highly myopic eye with a refractive error of − 20.0 diopters and an axial length of 30.69 mm. The image taken in 2001 shows a lacquer crack (black arrows) which is no longer detectable in the images taken in 2006 and 2011. Note: Marked enlargement of parapapillary gamma zone (green arrows); black asterisks: markings of a large choroidal vessel as fundus landmark

**Fig. 2:**
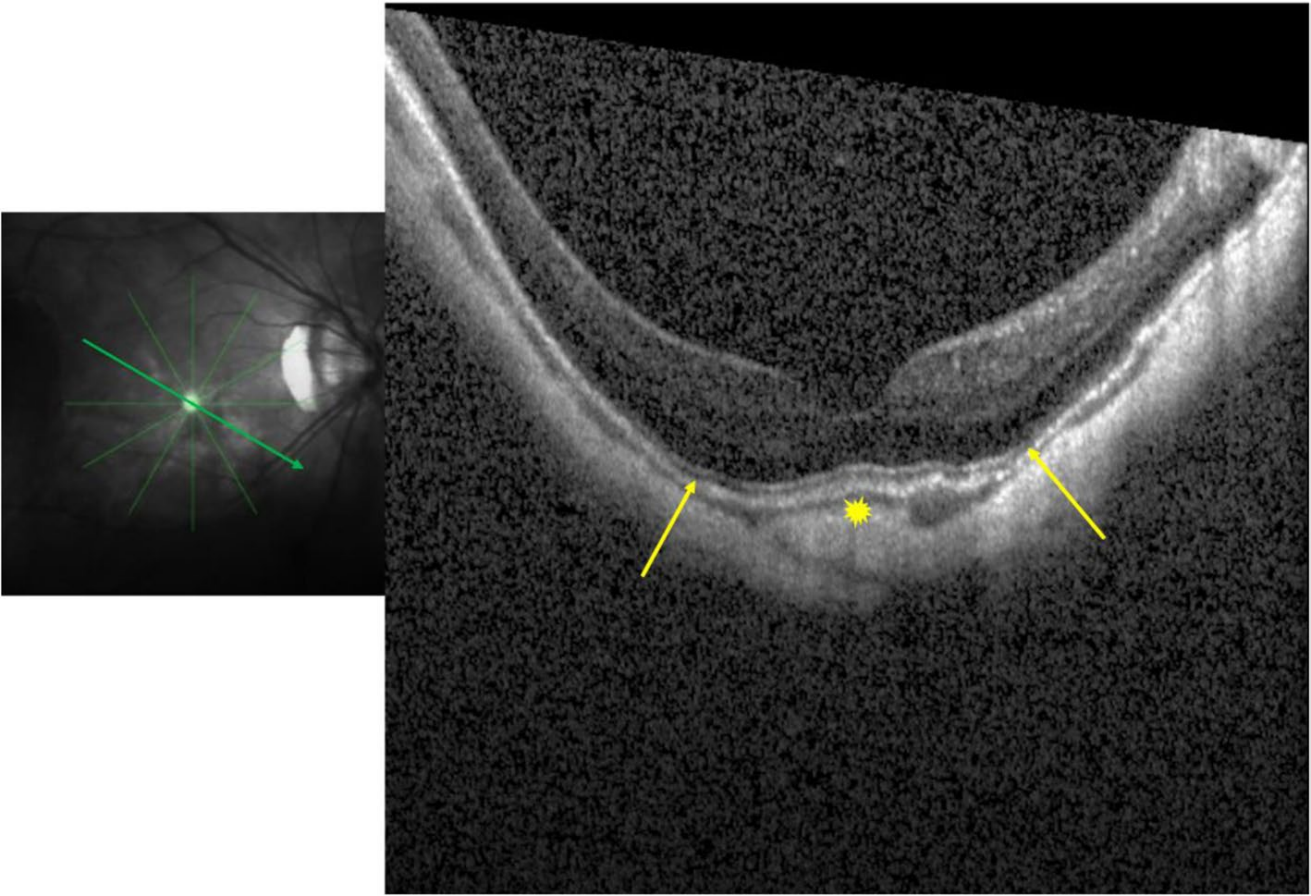
Optical coherence tomographic image taken in 2011 and showing a corrugated Bruch’s membrane (yellow arrows) besides a slightly dome-shaped macula (yellow asterisk), in the absence of any defect in Bruch’s membrane

A 56-year-old woman participating in the Beijing Eye Study 2001, 2006 and 2011, showed a LC, a vertically oval shaped optic disc, and parapapillary gamma zone on the fundus photograph taken in 2001 (Fig. 1a). Due to cortical-nuclear cataract, her best corrected visual acuity (BCVA) was 0.4 (20/50 Snellen) with a refractive error of − 19.0 diopters (spherical equivalent), and the intraocular pressure (IOP) was 10 mm Hg. When re-examined in 2006, the fundus photograph revealed an enlarged gamma zone, while the LC was no longer detectable (Fig. 1b). BCVA had remained unchanged, refractive error (spherical equivalent) was − 20.0 diopters, and IOP measured 17 mmHg. At the last examination performed in 2011, nuclear cataract had markedly progressed, BCVA had deteriorated to 0.2 (20/100 Snellen), and IOP was 11 mmHg. Refractive error was − 20.0 diopters and axial length measured 30.69 mm. As in 2006, the LC was not visible, while gamma zone had further enlarged (Fig. 1c). On optical coherence tomographic (OCT) images taken in 2011, a defect of the BM or RPE in the region of the former LC was not visible (Fig. 2). BM was corrugated suggesting a decreased BM strain [5]. The gamma zone enlargement occurred in a direction perpendicular to the direction of the former LC (Fig. 1a-c). In addition, the distance between the optic disc and choroidal vessels (as marked on Fig. 1) increased during the study period suggesting a change in the position of the large choroidal vessel in relationship to the large retinal vessels and the optic disc.

## Discussion and conclusions

The observation suggest that a LC can decrease in width (so that it is no longer detectable upon ophthalmoscopy and upon OCT), in temporal association with an enlargement of gamma zone. It fits with the notion that an enlargement of the BMO (i.e., enlarging gamma zone) may lead to a relaxation of the BM strain and subsequently to a decrease in the width of the LC. The more oval than circular enlargement of BMO (Fig. 1) may be due to a combined effect of a BMO enlargement and a shift of the BMO and the retinal layers in relationship to the scleral layer (including lamina cribrosa) as also shown and discussed previously in the Boramae Myopia Cohort Study [6]. In a similar manner, the change in the position of the large choroidal vessels in relationship to the large retinal vessels and the optic disc suggests a shifting of the large choroidal vessels in relationship to the inner layers, as also reported previously for prematurely born infants and in highly myopic adults [7, 8]. It again fits with the notion of a general shifting of the various layers of the choroid, BM, and retina, in relative and absolute terms, during the process of axial elongation [3, 4, 6–9].

## Data Availability

Upon reasonable request to the corresponding authors.

## Abbreviations

BCVA: Best corrected visual acuity
BM: Bruch’s membrane
BMO: Bruch’s membrane opening
IOP: Intraocular pressure
LCs: Lacquer cracks
OCT: Optical coherence tomography/ic
RPE: Retinal pigment epithelium
